# Identification of a Novel Myc-Regulated Gene Signature for Patients with Kidney Renal Clear Cell Carcinoma

**DOI:** 10.1155/2022/3487859

**Published:** 2022-12-26

**Authors:** Shengqiang Fu, Yifu Liu, Zhicheng Zhang, Ming Mei, Qiang Chen, Siyuan Wang, Xiaorong Yang, Ting Sun, Ming Ma, Wenjie Xie

**Affiliations:** ^1^Department of Urology, The First Affiliated Hospital of Nanchang University, Nanchang 330006, Jiangxi Province, China; ^2^Department of Day Ward, The First Affiliated Hospital of Nanchang University, Nanchang 330006, Jiangxi Province, China

## Abstract

Given that myc was known to be a cancer-causing gene in several cancers including kidney renal clear cell carcinoma (KIRC). We aimed to construct myc-regulated genes (MRGs)-based prognostic signature. We obtained the mRNA expression and clinical data of KIRC from The Cancer Genome Atlas (TCGA) database and MRGs from the Molecular Signature Database (MSigDB). Then, a prognostic signature consisting of 8 MRGs (IRF9, UBE2C, YBX3, CDKN2B, CKAP2L, CYFIP2, FBLN5, and PDLIM7) was developed by differential expression analysis, cox regression analysis, and least absolute shrinkage and selection operator (lasso) analysis. Patients with KIRC were divided into high- and low-risk groups based on risk scores of MRGs-based signatures. Patients in the high-risk group showed inferior clinical characteristics and survival. In addition, the risk score was an independent prognostic factor for KIRC, and the risk score=based nomogram displayed satisfactory performance to predict the survival of KIRC. The MRGs-based signature is also correlated with immune cell infiltration and the mRNA expression of important immune checkpoints (IDO2, PDCD1, LAG3, FOXP3, and TIGIT). The tumor mutation burden (TMB) landscape between the high- and low-risk groups showed higher levels of TMB in the high-risk group than in the low-risk group and that higher levels of TMB predicted a poorer prognosis in KIRC. Furthermore, patients with KIRC in the high-risk group are more likely to experience immune escape. At last, we found patients with KIRC in the high-risk group were more sensitive to several chemotherapy drugs such as sunitinib, gefitinib, nilotinib, and rapamycin than patients with KIRC in the low-risk group. Our study successfully constructed and validated an MRGs-based signature that can predict clinical characteristics, prognosis, level of immune infiltration, and responsiveness to immunotherapy and chemotherapy drugs in patients with KIRC.

## 1. Introduction

Renal cell carcinoma (RCC) is global cancer that affects more than 300,000 people worldwide each year [1]. Among all RCCs, kidney renal clear cell carcinoma (KIRC) is the most common pathological subtype, accounting for more than 70% of all RCCs [2]. Although KIRC is common, its onset is insidious and the early clinical symptoms are mild or nonexistent, and once patients developed typical clinical symptoms such as hematuria, abdominal mass, and back pain, it often indicates that cancer has entered an advanced stage and the best time for treatment has been missed [3, 4]. In addition, KIRC is an invasive tumor that can often develop distant organ metastases [5–7]. Delay in the diagnosis of KIRC and susceptibility of KIRC to metastasis contribute to the unsatisfactory prognosis of patients. Therefore, the identification of prognostic signatures and customization of treatment options are necessary to allow a satisfactory prognosis for patients with KIRC.

Myc is one of the most extensively studied oncogenes and is closely correlated with the initiation, maintenance, and progression of several cancers [8]. It encodes a protein that functions primarily as a transcriptional regulator of genes involved in the regulation of several cellular processes such as cell growth, cell cycle, cell differentiation, apoptosis, angiogenesis, metabolism, and immune response [4, 9]. Dysregulation of the expression of myc has been detected in several types of cancer including KIRC, and studies have demonstrated that myc played an important role in the progression of KIRC [10–12]. In view of the important role played by myc in KIRC progression, a comprehensive analysis of genes regulated by myc was performed in this study.

In the present study, we identified differentially expressed myc-regulated genes (MRGs) and explored their potential functions. These MRGs were further screened, and we finally obtained 8 MRGs (IRF9, UBE2C, YBX3, CDKN2B, CKAP2L, CYFIP2, FBLN5, and PDLIM7) based on which we successfully constructed and validated a prognostic signature of KIRC. We uncovered the correlation of MRGs-based signature with the clinical characteristics and prognosis of KIRC. Based on the evidence that myc was associated with the regulation of immune responses in cancer [13–15], we also explored and unveiled the correlation of MRGs-based signature with immune cell infiltration, immune checkpoint expression, tumor mutation burden (TMB), and immune treatment response in KIRC. In the end, we also found that the MRGs-based signature was significantly correlated with the sensitivity of KIRC to chemotherapeutic agents.

## 2. Methods

### 2.1. Data Acquisition

The MRGs were derived from four datasets including DANG_MYC_TARGETS_DN GeneSets (31 genes), DANG_MYC_TARGETS_UP GeneSets (130 genes), DANG_REGULATED_BY_MYC_DN GeneSets (251 genes), and DANG_REGULATED_BY_MYC_UP GeneSets (68 genes) in the Molecular Signature Database v7.1 (MSigDB; https://www.gsea-msigdb.org/gsea/msigdb). The mRNA expression profiles and clinical data of KIRC samples and normal control samples were obtained from The Cancer Genome Atlas database (https://portal.gdc.cancer.gov/).

### 2.2. Identification of Differentially Expressed MRGs

Analysis of differential mRNA expression of MRGs between KIRC samples and normal control samples was performed by running the limma R package. Differentially expressed MRGs were defined as the difference in mRNA expression of MRGs in KIRC samples and normal control samples that met both |log 2 fold change (FC)| > 1 and false discovery rate (FDR) < 0.05.

### 2.3. Enrichment Analysis of MRGs

Gene Ontology (GO) analysis focuses on the molecular function, biological process, and cellular component of the gene product; Kyoto Encyclopedia of Genes and Genomes (KEGG) pathway analysis focuses on the metabolic pathways in which the gene product is involved. In order to explore which functions and metabolic pathways MRGs are mainly involved in, GO and KEGG analyses were performed by running cluster Profiler R package, org.Hs.eg.db R package, and enrichplot R package.

### 2.4. Establishment and Validation of MRGs-Based Prognostic Signature

Samples included in the construction and validation of the risk model were subject to the two criteria of [1] having complete mRNA expression of MRGs and [2] having a minimum overall survival (OS) time of no less than 30 days. The samples eligible for inclusion in the model construction and validation were randomly divided into training and validation sets in a 7 : 3 ratio by running the caret R package.

First, to identify MRGs associated with the prognosis of KIRC in the training cohorts, univariate cox regression was performed. To avoid overfitting of MRGs with the model, least absolute shrinkage and selection operator (lasso) regression was then performed to remove MRGs that overfitted with the model. Later, multivariate cox regression analysis was performed to select risk MRGs independently from other factors. The risk score for each KIRC sample can be estimated by the following formula:

(1) $$\begin{array}{c} Risk\;score=\left(Coef1*mRNA\;expression\;of\;gene1\right)\\ +\left(Coef2*mRNA\;expression\;of\;gene2\right)\\ +\ldots \\ +\left(Coefn*\;mRNA\;expression\;of\;gene\;n\right)\text{.} \end{array}$$

The “Coef” is the coefficient in the lasso regression model. The KIRC samples in the training sets can be divided into high- and low-risk groups based on the median value of risk scores for all KIRC samples in the training sets. Principal component analysis (PCA) was performed to downscale this multigene signature model. The Kaplan–Meier survival analysis and time-dependent receiver operating characteristic (ROC) analysis were then performed to assess the ability of this signature in predicting the prognosis of KIRC. To evaluate the reliability and stability of the signature, the same analysis was performed on the test set and the total set. The “glmnet,” “survival,” “survminer,” and “time ROC” R packages were applied to perform these analyses.

### 2.5. Establishment of Risk Score-Based Nomogram

The correlation of MRGs-based signatures with the clinical characteristics of KIRC samples was first explored. Next, Cox regression analysis was performed to analyze whether the risk score of MRGs-based signature was an independent prognostic factor for the KIRC sample. A nomogram predicting the overall survival (OS) of the KIRC sample at 1, 2, and 3 years was constructed based on the independent factors of KIRC. Then, calibration curves were plotted to evaluate the performance of the nomogram in predicting KIRC for 1, 2, and 3 years of OS. The “survival” and “rms” R packages were used to perform these analyses.

### 2.6. Immune Analysis of MRGs-Based Signature

Given the importance of the tumor microenvironment (TME) in tumorigenesis and progression, the ESTIMATE algorithm was applied to analyze the level of infiltration of two important components of the TME, stromal cells, and immune cells. Then, the TIMER, CIBERSORT, CIBERSORT-ABS, QUANTISEQ, MCPCOUNTER, XCELL, and EPIC algorithms were applied to assess differences in immune cell infiltration in high- and low-risk groups, respectively. Next, Differences in immune cell abundance and differences in the enrichment of immune-related functions in high- and low-risk groups were revealed by the ssGSEA enrichment analysis. Furthermore, differences in the mRNA expression of immune checkpoints between high- and low-risk groups and correlations of risk scores with immune checkpoints were analyzed.

### 2.7. Analysis of TMB and TIDE Based on MRGs Signature

The TMB in KIRC was evaluated based on this formula: TMB = (total count of variants)/(the whole length of exons). We analyzed the respective mutational profiles of the KIRC samples in the high- and low-risk groups by applying the Maftools R package. Differences in TMB between KIRC patients in high- and low-risk groups, differences in Kaplan–Meier survival for KIRC patients with high and low levels of TMB, and differences in Kaplan–Meier survival across subgroups stratified by H−TMB + high risk, H−TMB + low risk, L−TMB + high risk and L−TMB + low risk were further analyzed by applying the limma, ggpubr, survival, and survminer R packages.

In addition, the difference in the therapeutic efficacy of immune checkpoint inhibitors between the high- and low-risk groups was obtained by calculating the difference in tumor immune dysfunction and exclusion (TIDE) scores in the TIDE database (http://tide.dfci.harvard.edu/) between the high- and low-risk groups.

### 2.8. Drug Sensitivity Analysis

To further investigate the sensitivity of KIRC to common chemotherapeutic drugs in the high- and low-risk groups, we searched the Genomics of Drug Sensitivity in Cancer (GDSC, http://www.cancerRxgene.org) database combined with the application of the R package to compare the differences in half-maximal inhibitory concentration (IC50) values.

### 2.9. Statistical Analyses

All statistical analyses in the present study were performed by R (version 4.0.0). Wilcoxon rank-sum test was used to compare gene expression differences between KIRC samples and normal control samples. As for the correlation analysis, the Pearson correlation coefficient was adopted when the data met the three conditions of continuous variables, normal distribution, and linear relationship, otherwise, the Spearman correlation coefficient was used. *P* values less than 0.05 were considered statistically different.

## 3. Results

### 3.1. Identification of the Differentially Expressed MRGs

**Figure 1:**
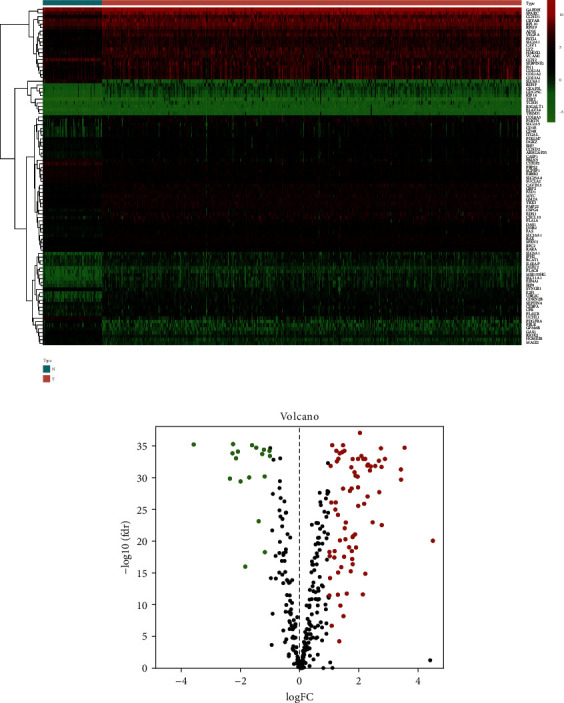
Differentially expressed MRGs in the TCGA database. (a) Heatmap showed MRGs differentially expressed between KIRC samples and normal samples. Red represented the upregulation of MRGs expression and green represented downregulation of MRGs expression. (b) Volcano displayed differentially expressed MRGs. FC: fold change, fdr: false discovery rate, KIRC: kidney renal clear cell carcinoma, MRGs: myc-regulated genes, TCGA: The Cancer Genome Atlas.

We obtained 480 MRGs from the MSigDB database, and the mRNA expression levels of MRGs were obtained by comparing with RNA-seq data downloaded from the TCGA database including 539 KIRC samples and 72 normal control samples. By differential expression analysis, we finally successfully identified 94 differentially expressed MRGs including 18 downregulated MRGs and 76 upregulated MRGs based on the screening criteria of |log 2 FC| > 1 and FDR < 0.05. The results of this differential expression analysis are presented in the form of a heat map and a volcano map, respectively (Figures 1(a), 1(b)).

### 3.2. Functional Enrichment Analysis of Differentially Expressed MRGs

**Figure 2:**
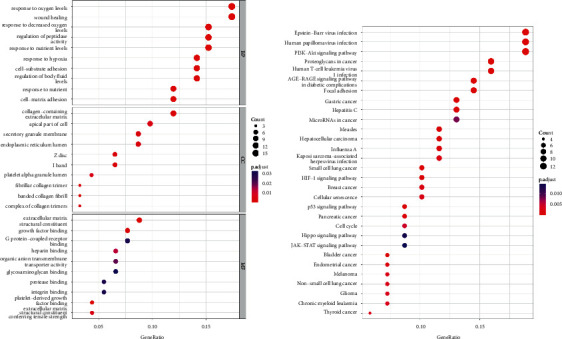
Enrichment analysis of differentially expressed MRGs. (a) GO analysis including BP, CC, and MF categories of differentially expressed MRGs. (b) KEGG pathway analysis of differentially expressed MRGs. BP: biological process, CC: cellular component, GO: gene ontology, KEGG: Kyoto Encyclopedia of Genes and Genomes, MF: molecular function, MRGs: myc-regulated genes.

To uncover the potential molecular functions and mechanisms of differentially expressed MRGs, we performed GO and KEGG pathway enrichment analysis on differentially expressed MRGs. According to the low to high *p* values and high to low correlation coefficients, we found that the top three GO terms in biological process (BP) were response to oxygen levels, wound healing, and response to decreased oxygen levels. In the cellular component (CC) category, collagen−containing extracellular matrix, apical part of the cell, and secretory granule membrane occupied the top three GO terms. Extracellular matrix structural constituent, growth factor binding, and G protein−coupled receptor binding were the top three GO terms in the molecular function (MF) category. The results of the KEGG pathway enrichment analysis showed that differentially expressed MRGs were closely associated with Epstein−Barr virus infection, human papillomavirus infection, and PI3K−Akt signaling pathway. The results of GO and KEGG pathway enrichment analysis are shown together in Figure 2.

### 3.3. Construction and Validation of a Prognostic MRGs-Based Signature

**Figure 3:**
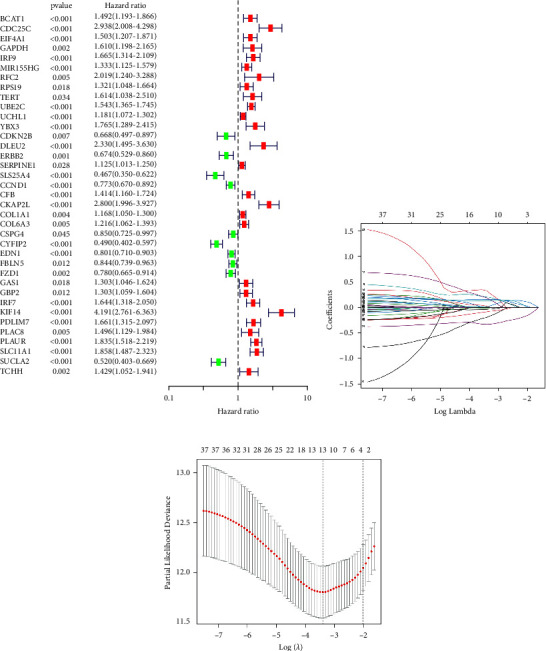
Construction of MRGs-based signature. (a) Univariate regression analysis to identify MRGs associated with the prognosis of KIRC. (b) Lasso regression analysis to remove MRGs that overfitted with the model. (c) Tenfold cross-validation to obtain satisfactory parameters. KIRC: kidney renal clear cell carcinoma, Lasso: least absolute shrinkage and selection operator, MRGs: myc-regulated genes.

A total of 515 KIRC samples were included in the construction and validation of the risk model. The 515 KIRC samples were next randomly divided into a training set of 361 samples and a test set of 154 samples in a ratio of 7 : 3. The results of the univariate Cox regression analysis in the training set showed that 37 MRGs were significantly associated with the prognosis of the KIRC sample (*p* < 0.05) (Figure 3(a)). Next, we successfully constructed a risk model consisting of 8 MRGs (IRF9, UBE2C, YBX3, CDKN2B, CKAP2L, CYFIP2, FBLN5, and PDLIM7) by performing the lasso regression analysis (Figures 3(b) and 3(c)). The list of genes and coefficients used to construct the risk model are shown in Table 1.

**Figure 4:**
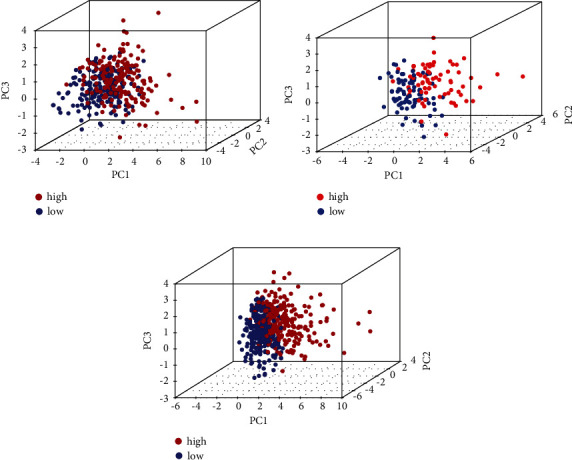
PCA analysis: (a) PCA analysis in the training set; (b) PCA analysis in the test set; (c) PCA analysis in the overall set. PCA: principal components analysis.

The risk score for the KIRC samples in the risk model was obtained according to the following formula: Risk Score = (mRNA expression of IRF9 *∗*0.223355169440029) − (mRNA expression of UBE2C *∗*0.223355169440029) + (mRNA expression of YBX3 *∗*0.480900005160846) − (mRNA expression of CDKN2B *∗*0.596863859303069) + (mRNA expression of CKAP2L *∗*0.953589914105029) − (mRNA expression of CYFIP2 *∗*0.470828182577709) − (mRNA expression of FBLN5 *∗*0.186711438630099) + (mRNA expression of PDLIM7 *∗*0.334470058632468). In this way, we can calculate the median value of risk scores for all KIRC samples in the model, and based on the median value, the KIRC samples can be divided into high-risk and low-risk groups. The results of PCA analysis showed a significant discrete trend in the three-dimensional plane for the high- and low-risk groups (Figure 4).

**Figure 5:**
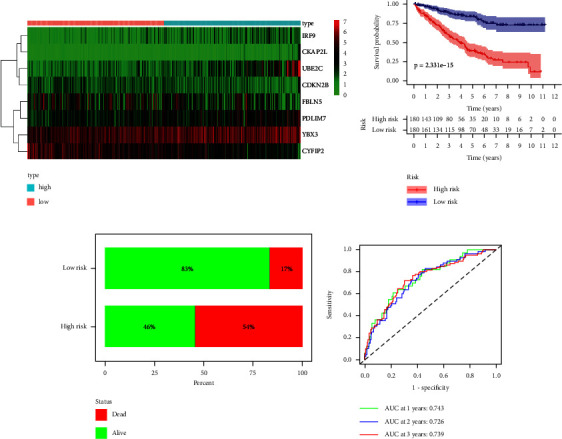
Validation of eight MRGs signature in the training test (*n* = 361). (a) Heatmap of 8 MRGs expressions. The color from green to red represents the expression level of MRGs from low to high. (b) Kaplan–Meier survival analysis results for the KIRC samples between high- and low-risk groups. (c) Comparison of survival status of KIRC samples between high- and low-risk groups. (d) The ROC curve of the risk score in predicting 1-year, 2-year, and 3-year survival times for the KIRC samples. KIRC: kidney renal clear cell carcinoma, MRGs: myc-regulated genes, ROC, receiver operating characteristic.

**Figure 6:**
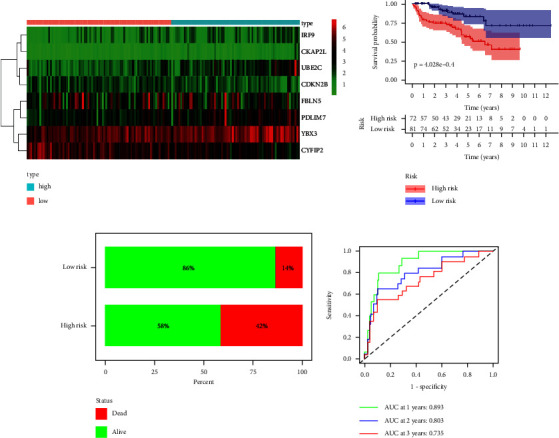
Validation of eight MRGs signature in the test set (*n* = 154). (a) Heatmap of 8 MRGs expressions. The color from green to red represents the expression level of MRGs from low to high. (b) Kaplan–Meier survival analysis results for the KIRC samples between high- and low-risk groups. (c) Comparison of survival status of KIRC samples between high- and low-risk groups. (d) The ROC curve of the risk score in predicting 1-year, 2-year, and 3-year survival times for the KIRC samples. KIRC: kidney renal clear cell carcinoma, MRGs: myc-regulated genes, ROC: receiver operating characteristic.

**Figure 7:**
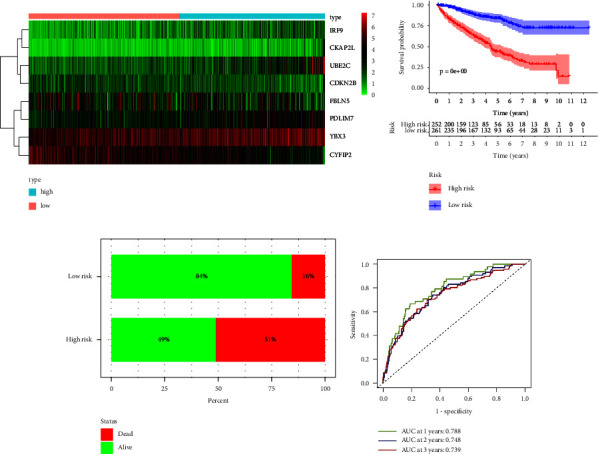
Validation of eight MRGs signature in the overall set (*n* = 515). (a) Heatmap of 8 MRGs expressions. The color from green to red represents the expression level of MRGs from low to high. (b) Kaplan–Meier survival analysis results for the KIRC samples between high- and low-risk groups. (c) Comparison of survival status of KIRC samples between high- and low-risk groups. (d) The ROC curve of the risk score in predicting 1-year, 2-year, and 3-year survival times for the KIRC samples. KIRC: kidney renal clear cell carcinoma, MRGs: myc-regulated genes, ROC: receiver operating characteristic.

The mRNA expression of the eight MRGs in the high- and low-risk groups was presented as a heatmap in Figure 5(a). There was a significant difference in OS between the high- and low-risk groups, as shown in Figure 5(b), where the Kaplan–Meier survival analysis between the high- and low-risk groups showed that OS was significantly lower in the high-risk group than in the low-risk group (*p*=2.331*e* − 15). Consistent with this, the survival status of the high-risk group (percentage of death status 54%) was also significantly worse than that of the low-risk group (percentage of death status 17%) (Figure 5(c)). The time-dependent ROC showed the accuracy of the risk score in predicting OS at 1, 2, and 3 years in the KIRC sample to be 0.743, 0.726, and 0.739, respectively (Figure 5(d)). The test set and the overall set were performed with the same analysis to verify the stability and reliability of the model constructed from the training set. MRGs incorporated into the constructed risk model exhibited expression levels consistent with the training set in both the test and total sets (Figures 5(a), 6(a), 7(a)). The differences in OS and survival status in the high- and low-risk groups in the test and total sets were consistent with those exhibited in the high- and low-risk groups in the training set (Figures 5(b), 5(c), 6(b), 6(c), 7(b), and 7(c)). Time-dependent ROC analysis showed that the accuracy of the risk score in predicting the 1-year, 2-year, and 3-year OS of the KIRC sample in the test set was 0.893, 0.803, and 0.735, respectively (Figure 6(d)), and in the overall set was 0.788, 0.748, and 0.739, respectively (Figure 7(d)). The risk scores for both the test and total sets demonstrate similar accuracy in predicting the 1-year, 2-year, and 3-year OS of the KIRC samples as the risk scores for the training set.

### 3.4. Correlation between MRGs-Based Signature and Clinical Characteristics

**Figure 8:**
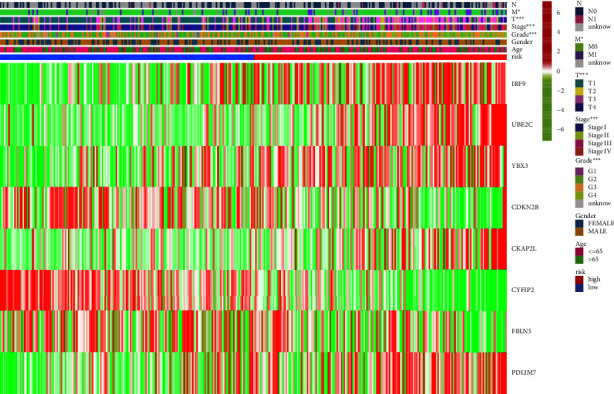
Correlation analysis of the MRGs-based signature and clinical characteristics of KIRC samples. KIRC: kidney renal clear cell carcinoma, MRGs: myc-regulated genes. (^*∗*^*P* < 0.05; ^*∗∗∗*^*P* < 0.001).

We also explored the correlation between MRGS-based signatures and clinical characteristics of the KIRC samples. The heatmap (Figure 8) showed that the mRNA expression levels of IRF9, UBE2C, YBX3, CKAP2L, and PDLIM7 were higher in the high-risk group than in the low-risk group, while the mRNA expression levels of CDKN2B, CYFIP2, and FBLN5 were lower in the high-risk group than in the low-risk group. In addition, an important finding was that samples in the high-risk group possessed inferior clinical characteristics including higher grade, stage, T-staging, and M-staging in the TNM staging system than those in the low-risk group.

**Figure 9:**
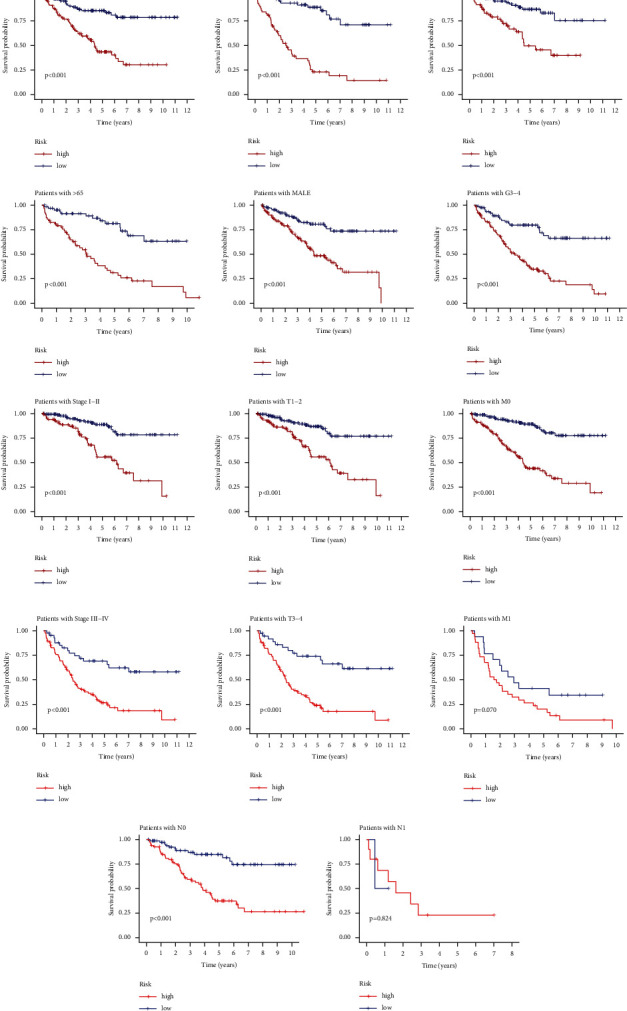
Kaplan–Meier survival analysis of high- and low-risk KIRC samples between subgroups stratified by age, gender, grade, stage, T stage, M stage, and N stage. KIRC: kidney renal clear cell carcinoma.

Furthermore, the relationship between MRGs-based signature and the prognosis of KIRC in each clinical subgroup stratified by age (≤65 years or >65 years), gender (female or male), grade (G1 + 2 or G3 + 4), stage (stage I + II or III + IV), T stage (T1 + 2 or T3 + 4), M stage (M0 or M1), and N stage (N0 or N1) was analyzed. The Kaplan–Meier analysis showed that higher risk scores were correlated with worse prognosis in multiple subgroups including age ≤65 years or >65 years, female or male, G1 + 2 or G3 + 4, Stage I + II or III + IV, T1 + 2 or T3 + 4 stage, M0 or M1 stage, and N0 stage compared to lower risk scores (Figure 9). It should be noted that in the N1 subgroup (Figure 9), the risk score was not significantly associated with the prognosis of the KIRC sample, which may be due to the small number of KIRC samples in the N1 subgroup.

### 3.5. Construction of the Nomogram

**Figure 10:**
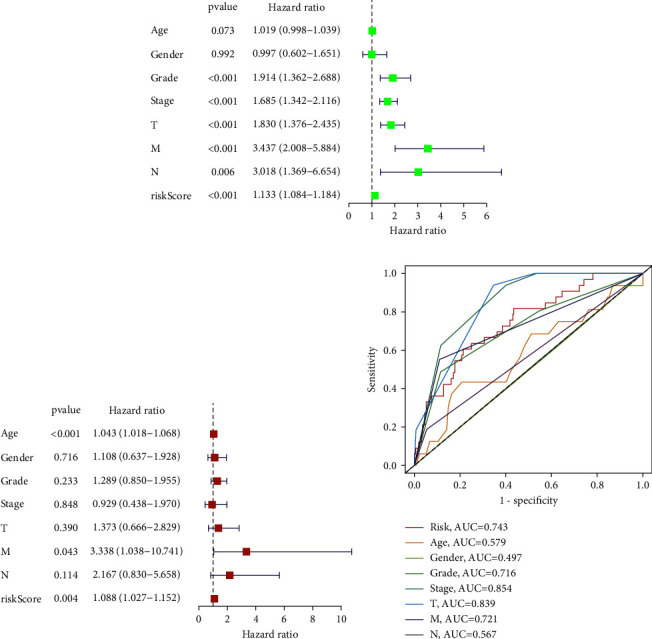
MRGs-based risk signature was an independent prognostic factor for the KIRC samples. (a) Univariate cox regression analysis of risk scores and clinical characteristics to identify factors associated with the prognosis of KIRC. (b) Multivariate cox regression analysis of risk scores and clinical characteristics was performed to identify factors that could independently influence the prognosis of KIRC. (c) ROC curves for risk score and clinical characteristics to predict prognosis of KIRC. KIRC: kidney renal clear cell carcinoma, MRGs: myc-regulated genes, ROC: receiver operating characteristic.

We included the clinical characteristics including age, gender, grade, stage, T stage, M stage, N stage, and risk score of the KIRC sample in univariate and multivariate Cox regression analysis models. The results of univariate Cox regression analysis showed age (HR 1.019; 95% CI 0.998–1.039; *p*=0.073), grade (HR 1.914; 95% CI 1.362–2.688; *p* < 0.001), stage (HR 1.685; 95% CI 1.342–2.116; *p* < 0.001), T stage (HR 1.830; 95% CI 1.376–2.435; *p* < 0.001), M stage (HR 3.437; 95% CI 2.008–5.884; *p* < 0.001), N stage (HR 3.018; 95% CI 1.369–6.654; *p*=0.006), and risk score (HR 1.133; 95% CI 1.084–1.184; *p* < 0.001) were significantly correlated with the prognosis of the KIRC samples (Figure 10(a)). However, the results of the multivariate Cox regression analysis showed that only age (HR 1.043; 95% CI 1.018–1.068; *p* < 0.001), M stage (HR 3.338; 95% CI 1.038–10.741; *p*=0.043), and risk score (HR 1.088; 95% CI 1.027–1.152; *p*=0.004) were independent prognostic factors for the KIRC samples (Figure 10(b)). In addition, we compared the accuracy of risk and traditional clinical characteristics in predicting prognosis in the KIRC. The results showed that the accuracy of risk in predicting one-year OS in the KIRC sample was 0.743, which was only less accurate than that of the stage (AUC = 0.854) and T stage (AUC = 0.839), and better than most of the clinical variables such as age (AUC = 0.579), gender (AUC = 0.497), grade (AUC = 0.716), M stage (AUC = 0.721), and N stage (AUC = 0.567) (Figure 10(c)).

**Figure 11:**
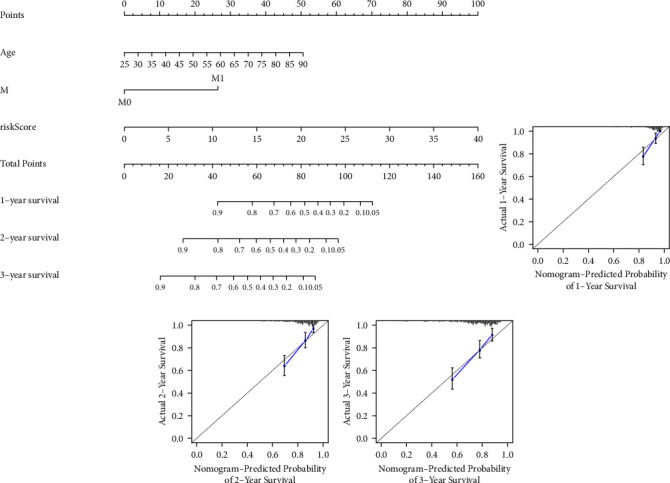
Construction of a nomogram: (a) Construction of nomogram for predicting 1-year, 2-year, and 3-year OS of KIRC based on MGRs signature. (b) Calibration curves for predicting the 1-year, 2-year, and 3-year OS of KIRC. KIRC: kidney renal clear cell carcinoma, MRGs: myc-regulated genes, OS: overall survival.

To further predict the prognosis of KIRC, we constructed an MRGs-based nomogram. As shown in Figure 11(a), the nomogram predicted the OS of the KIRC sample at 1, 2, and 3 years, respectively. The C-index of 0.747 implied that the nomogram had moderate accuracy in predicting KIRC 1-year, 2-year, and 3-year OS. In addition, the calibration curves also showed that the 1-year, 2-year, and 3-year survival probabilities of the patients with KIRC predicted by the nomogram were consistent with the actual survival probabilities of the patients (Figures 11(b)–11(c)).

### 3.6. Difference of Immune Characteristics of MRGs-Based Signature

**Figure 12:**
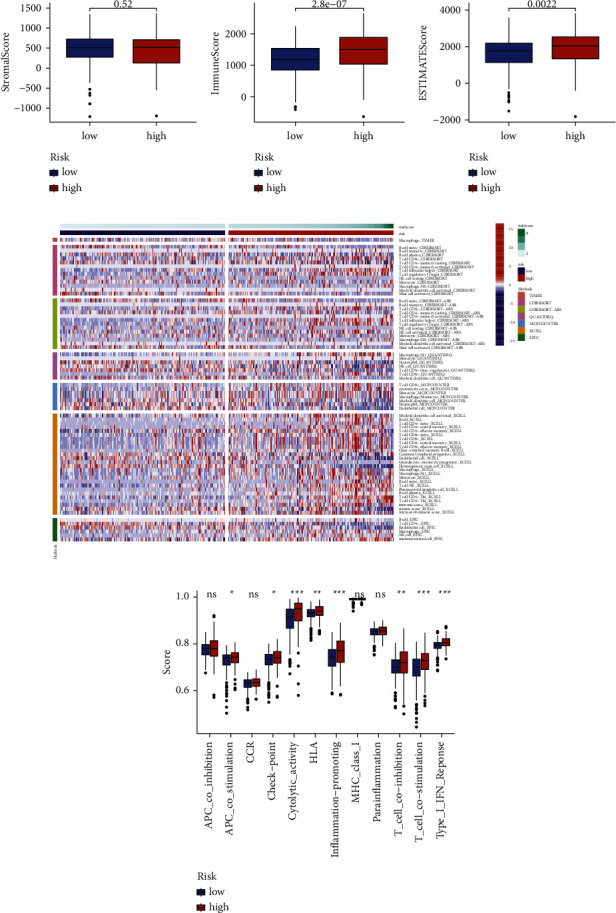
Analysis of the immune landscape between high- and low-risk groups. (a) Differential analysis of the tumor microenvironment between high- and low-risk groups by ESTIMATE algorithm. (b) Differences in the level of immune cell infiltration between high- and low-risk groups by CIBERSORT, QUANTISEQ, MCPcounter, XCELL, CIBERSORT-ABS, TIMER, and EPIC algorithms. (c) Differences in enrichment analysis of immune function between high- and low-risk groups by ssGSEA algorithm. TME: tumor microenvironment. (^*∗*^*P* < 0.05; ^*∗∗*^*P* < 0.01; ^*∗∗∗*^*P* < 0.001).

To explore the correlations between MRGs-based signature and immune characteristics in the TME, we performed a series of analyses including the correlation analyses between MRGs-based signature and immune cells, immune checkpoints, and immune escape. The results of stromal cell and immune cell infiltration analysis in TME showed that higher levels of immune cell infiltration in the high-risk group than in the low-risk group in TME (*p*=2.8*e* − 07) (Figures 12(a)–12(c)). The results of immune cell infiltration analysis based on TIMER, CIBERSORT, CIBERSORT-ABS, QUANTISEQ, MCPCOUNTER, XCELL, and EPIC algorithms showed that the risk score of MRGs-based signature was closely correlated with the infiltration of immune cells (Figure 12(d)). Enrichment analysis of immune-related functions in the high- and low-risk groups showed that APC_co_stimulation, checkpoint, cytolytic_activity, HLA, Inflammation−promoting, T_cell_coinhibition, T_cell_costimulation, and Type_I_IFN_response were more active in the high-risk group than in the low-risk group (Figure 12(e)).

**Figure 13:**
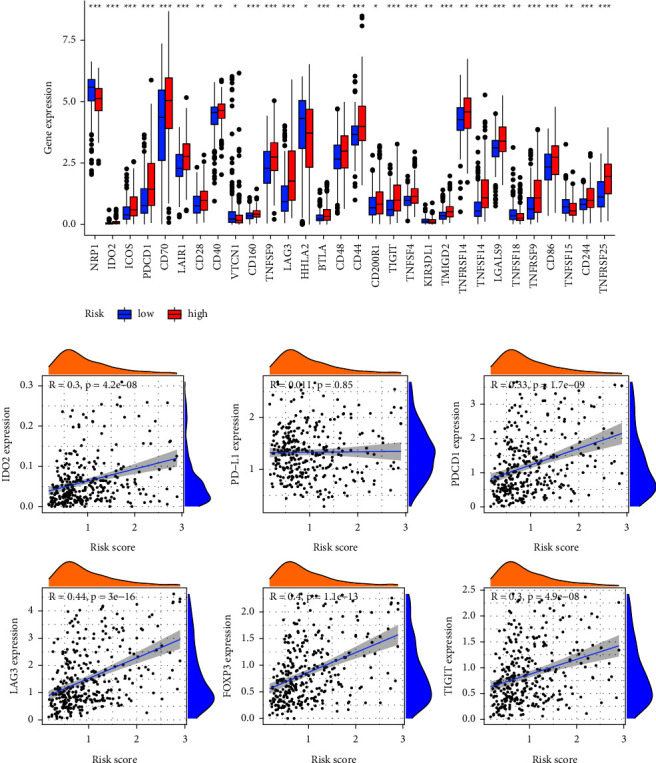
Relationship between the immune checkpoint and MRGs-based signature. (a) Differences in expression of immune checkpoints between high- and low-risk groups. (b) Correlation analysis of risk scores with immune checkpoint expression. MRGs: myc-regulated genes. (^*∗*^*P* < 0.05; ^*∗∗*^*P* < 0.01; ^*∗∗∗*^*P* < 0.001).

We further explored the differences in the expression of immunosuppressive molecules in the high- and low-risk groups. The results showed that most of the immune checkpoint molecules in the high-risk group, including ICOS, PDCD1, CD70, LAIR1, CD28, CD40, CD160, TNFSF9, LAG3, BTLA, CD48, CD44, CD200R1, TIGIT, TNFSF4, TMIGD2, TNFRSF14, LGALS9, TNFRSF9, CD86, CD244, and TNFRSF25, with higher expression levels than those in the low-risk group (Figure 13(a)). We also analyzed the correlation between the expression of several key immune checkpoints and risk scores. The results showed that IDO2 (*R* = 0.3, *p*=4.2*e* − 08), PDCD1 (*R* = 0.33, *p*=1.7*e* − 09), LAG3 (*R* = 0.44, *p*=3*e* − 16), FOXP3 (*R* = 0.4, *p*=1.1*e* − 13), and TIGIT (*R* = 0.3, *p*=4.9*e* − 08) were positively correlated with the risk score (Figure 13(b)). The results of the immune infiltration analysis showed the immunosuppressed status of the KIRC samples in the high-risk group.

### 3.7. Difference between TMB and TIDE Based on MRGs Signatures

**Figure 14:**
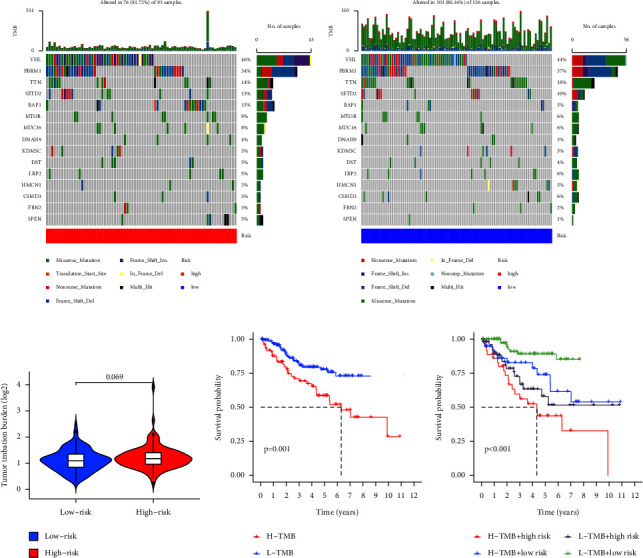
Correlation analysis of prognostic signature with TMB. (a) The mutational landscape between high- and low-risk groups. (b) Differences in the levels of TMB between high- and low-risk groups. (c) Differences in Kaplan–Meier survival between high and low levels of TMB. (d) Differences in Kaplan–Meier survival between subgroups stratified by H−TMB + high risk, H−TMB + low risk, L−TMB + high risk, and L−TMB + low risk. TMB: tumor mutation burden.

We further explored mutational characteristics between high- and low-risk groups. The TMB landscape in the high- and low-risk groups showed that the frequency of mutations in SETD2, BAP1, and MTOR was higher in the high-risk group than in the low-risk group (Figure 14(a)). In addition, the results of the correlation analysis between risk and TMB levels showed that the levels of TMB in the high- and low-risk samples were close to being statistically different (*p*=0.069) (Figure 14(b)). The Kaplan–Meier survival analysis showed higher levels of TMB were associated with a worse prognosis for KIRC patients (*p*=0.001) (Figure 14(c)). Results of survival analysis for subgroups stratified by H−TMB + high risk, H−TMB + low risk, L−TMB + high risk, and L−TMB + low risk showed that patients with high levels of TMB and high risk have the worst prognosis, while those with low levels of TMB and low risk have the best prognosis (*p* < 0.001) (Figure 14(d)).

**Figure 15:**
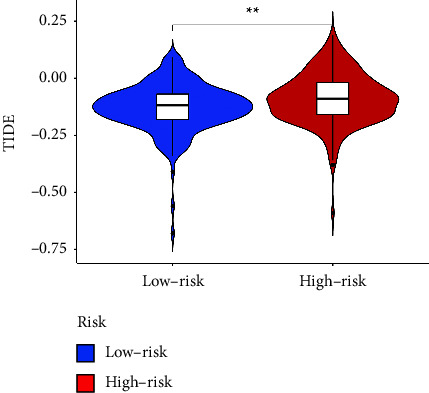
Differences in TIDE scores between high- and low-risk groups. TIDE: tumor immune dysfunction and exclusion. (^*∗∗*^*p* < 0.01).

Immune checkpoint inhibition therapy has become a hot treatment modality for cancer [16]. The TIDE score is gaining popularity because it is more accurate than a single biomarker for predicting the efficacy of immune checkpoint blockade for cancer [17]. We analyzed the difference in TIDE scores between the high- and low-risk groups, and the results showed that the TIDE scores were significantly higher in the high-risk group than in the low-risk group (*p* < 0.01) (Figure 15).

### 3.8. Analysis of MRGs-Based Signature and Chemotherapy Drug Sensitivity

**Figure 16:**
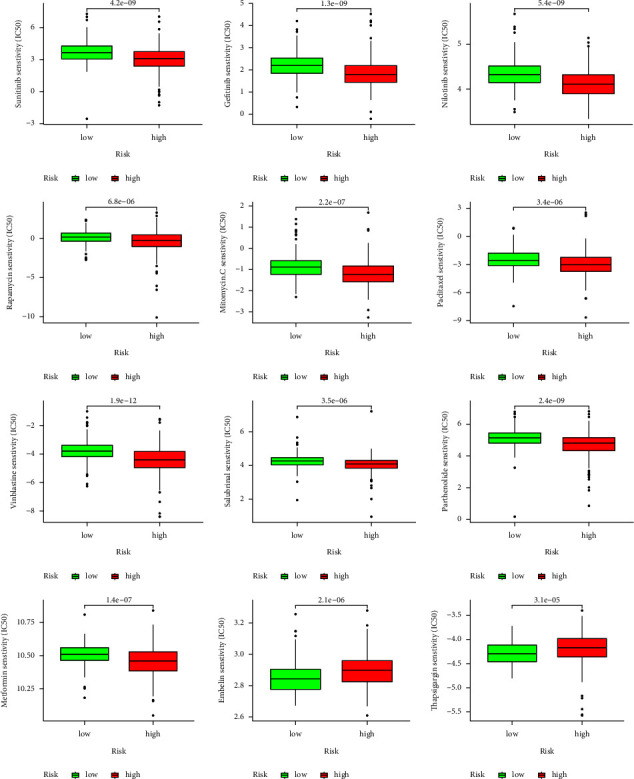
Differential analysis of IC50 value of sunitinib, gefitinib, nilotinib, rapamycin, mitomycin.C, paclitaxel, vinblastine, salubrinal, parthenolide, metformin, embelin, and thapsigargin between high- and low-risk groups. IC50: half-maximal inhibitory concentration.

The results of the correlation analysis between MRGs-based signature and chemotherapeutic drug sensitivity showed that the patients in the high-risk group had lower IC50 values compared to patients in the low-risk group for sunitinib, gefitinib, nilotinib, rapamycin, mitomycin.C, paclitaxel, vinblastine, salubrinal, parthenolide, and metformin. However, the IC50 values for embelin and thapsigargin were lower in the low-risk group than in the high-risk group (Figure 16).

## 4. Discussion

In this study, we identified 94 MRGs and analyzed the potential functions of these MRGs. The results of the GO and KEGG pathway enrichment analysis displayed the complexity of the function of the MRGs signature, suggesting the application potential of the MRGs signature. Combining cox regression analysis and lasso regression analysis we successfully constructed a prognostic signature consisting of eight MRGs (IRF9, UBE2C, YBX3, CDKN2B, CKAP2L, CYFIP2, FBLN5, and PDLIM7). The results of PCA and t-SNE analysis displayed that the signature received good dimensionality reduction, which implied the reliability of the risk model. The results of the prognostic analysis of the samples in the test and total sets further increase the credibility of our risk model constructed using the training set. The results of the correlation analysis between MRGs-based signature and clinical characteristics revealed that patients in the high-risk group were significantly associated with malignant clinical characteristics such as high grade, high stage, high T stage, and high M stage, which implied a poor prognosis for patients. In addition, the results of the Kaplan–Meier survival analysis for subgroups showed significant differences in OS between patients in high- and low-risk groups across multiple subgroups. The results of these analyses implied the satisfactory performance of MRGs-based signatures to predict the prognosis of KIRC. The results of univariate and multivariate Cox regression analyses demonstrated that risk score could independently predict the prognosis of patients with KIRC, and risk score-based nomogram and calibration curves suggested satisfactory accuracy of MRGs-based signature in predicting the prognosis of KIRC patients.

The TME is highly complex, and the study of TME can help provide new ideas for the treatment of tumors [18]. Stromal cells and immune cells are two important cell members of the TME [19]. Stromal cells have been demonstrated to be closely associated with the growth, metastasis, drug resistance of several cancers [20–22], and immune cells, depending on their type, can play an important role in fighting tumors and in promoting tumor progression or immune escape, respectively [23, 24]. Analytical results of TME based on MRGs signature showed that higher risk scores were associated with higher levels of immune cell infiltration, with no significant correlation between stromal cells and risk scores. This suggested that MRGs-based signature may affect the prognosis of KIRC by influencing the immune cell landscape in TME. The higher level of immune cell infiltration and immune function enrichment scores in the high-risk group further suggested that the infiltration of immune cells in TME may have contributed to tumor progression. The results of the analysis of the difference in the expression of immune checkpoints between high- and low-risk groups implied an immunosuppressed status in the KIRC samples from the high-risk group. Studies have shown that IDO2 was associated with B-cell immunity and can regulate T-cell-related immunity by affecting B-cell intrinsic mechanisms [25, 26]. PDCD1, also known as PD1, were known as programmed death ligands and receptors, respectively, and its combination with PD-L1 allows tumor cells to evade the body's immune surveillance [27]. FOXP3 is an important marker molecule of Tregs that directly or indirectly regulates the activity and function of Tregs, and changes in its protein levels have been shown to be closely associated with a variety of human diseases including tumorigenesis and metastasis [28–31]. LAG-3 was demonstrated to negatively regulate T-cell function, and antibodies to LAG-3 could relieve the inhibition of T-cell function by Tregs [32]. TIGIT is expressed in various T-cell subsets (CD4+ T, CD8+ T, Tregs) and can suppress the body's innate and adaptive immunity through various mechanisms and is considered a promising target for immunotherapy [33]. Takamatsu et al. demonstrated that profiles of LAG-3, TIM-3, and TIGIT were valuable for predicting the prognosis and TME of KIRC [34]. The significant positive correlation of risk scores with these key immune checkpoints further confirmed that upregulation of the expression of immune checkpoint in the KIRC patients from a high-risk group may be responsible for their poor prognosis.

With the widespread use of high-throughput sequencing technology, TMB has become a marker for predicting the response to immune checkpoint blockade in several types of cancer [35]. Although, in general, higher levels of TMB in cancer can lead to more infiltration of CD8+ T cells and thus contribute to a better prognosis by exerting an antitumor effect, KIRC has been shown to be cancer that challenged conventional thinking about cancer immunology based on this evidence that KIRC has a modest mutation burden but is responsive to immunotherapy and higher CD8+ T-cell infiltration is usually associated with a poorer prognosis [36, 37]. Our study displayed that patients with KIRC in the high-risk group had higher levels of TMB than those with KIRC in the low-risk group and that the higher the TMB the worse the prognosis for KIRC, which further validated the unusual relationship between the TMB and immunology in KIRC. SETD2, BAP1, and MTOR possessed higher mutation frequencies in the high-risk group than in the low-risk group implying that these three cancer-driven mutations may promote the progression of KIRC. The difference in TIDE scores between KIRC in the high- and low-risk groups reflected the effectiveness of immune checkpoint blockade, with higher TIDE scores indicating poorer immune checkpoint blockade, and higher TIDE scores for KIRC patients in the high-risk group than those in the low-risk group suggesting that patients in the high-risk group were more likely to experience immune escape, which was consistent with our analysis results that KIRC patients in the high-risk group had a worse prognosis than those in the low-risk group.

Of these MRGs, some have been shown to be associated with KIRC progression. IRF9 was thought could predict the prognosis and immune characteristics of KIRC as a member of a multigene signature [38, 39]. UBE2C was shown to be closely associated with the proliferation and invasion of KIRC and could predict the immune characteristics and prognosis of KIRC [40–42]. Jafri et al. identified germline CDKN2B mutations as a novel causative agent of familial KIRC, suggesting an important role for CDKN2B in the initiation of KIRC [43]. CYFIP2 was predicted to be downregulated in KIRC and downregulation of expression of CYFIP2 was associated with poor prognosis of KIRC [44]. In a rat model, FBLN5 was found to be associated with the metastasis of KIRC [45]. Other MRGs were not reported in KIRC, so our future studies will focus on these MRGs.

The results of the analysis of differences in sensitivity to chemotherapeutic agents between high- and low-risk groups revealed that patients in the high-risk group benefited more from treatment with sunitinib, gefitinib, nilotinib, rapamycin, mitomycin.C, paclitaxel, vinblastine, salubrinal, parthenolide, and metformin, while patients in the low-risk group benefited more from treatment with embelin and thapsigargin. These findings provide a theoretical basis for individualized pharmacological treatment of patients with KIRC.

The lack of prognostic information in all KIRC-related datasets in the GEO database resulted in our inability to validate our results with an independent external dataset, which is the main limitation of this study. Further studies need to be performed to explore the molecular mechanisms and biological functions of MRGs-based signatures.

## 5. Conclusion

In this study, a prognostic signature consisting of 8 MRGs (IRF9, UBE2C, YBX3, CDKN2B, CKAP2L, CYFIP2, FBLN5, and PDLIM7) in KIRC was successfully constructed and validated. The MRGs-based signature can predict the clinical characteristics, prognosis, immune characteristics, and sensitivity to chemotherapeutic agents of patients with KIRC and has the potential to be applied in the clinical setting.

## Figures and Tables

**Table 1:** The list of genes and coefficients in the prognostic signature.

Genes	Coef
IRF9	0.223355169440029
UBE2C	−0.225284628293333
YBX3	0.480900005160846
CDKN2B	−0.596863859303069
CKAP2L	0.953589914105029
CYFIP2	−0.470828182577709
FBLN5	−0.186711438630099
PDLIM7	0.334470058632468

## Data Availability

All raw data in this study were obtained from the GDC database (https://portal.gdc.cancer.gov/), TIDE database (http://tide.dfci.harvard.edu/), and GDSC database (http://www.cancerRxgene.org).
